# Odorant Binding Causes Cytoskeletal Rearrangement, Leading to Detectable Changes in Endothelial and Epithelial Barrier Function and Micromotion

**DOI:** 10.3390/bios13030329

**Published:** 2023-02-28

**Authors:** Theresa M. Curtis, Annabella M. Nilon, Anthony J. Greenberg, Matthew Besner, Jacob J. Scibek, Jennifer A. Nichols, Janet L. Huie

**Affiliations:** 1 Department of Biological Sciences, SUNY Cortland, Cortland, NY 13045, USA; 2 Bayesic Research, LLC, Ithaca, NY 14850, USA; 3 Jan Biotech, Inc., Ithaca, NY 14850, USA

**Keywords:** odorant cell-based biosensor, electrical resistance, micromotion, cytoskeleton, olfactory receptor, barrier function

## Abstract

Non-olfactory cells have excellent biosensor potential because they express functional olfactory receptors (ORs) and are non-neuronal cells that are easy to culture. ORs are G-protein coupled receptors (GPCRs), and there is a well-established link between different classes of G-proteins and cytoskeletal structure changes affecting cellular morphology that has been unexplored for odorant sensing. Thus, the present study was conducted to determine if odorant binding in non-olfactory cells causes cytoskeletal changes that will lead to cell changes detectable by electric cell-substrate impedance sensing (ECIS). To this end, we used the human umbilical vein endothelial cells (HUVECs), which express OR10J5, and the human keratinocyte (HaCaT) cells, which express OR2AT4. Using these two different cell barriers, we showed that odorant addition, lyral and Sandalore, respectively, caused an increase in cAMP, changes in the organization of the cytoskeleton, and a decrease in the integrity of the junctions between the cells, causing a decrease in cellular electrical resistance. In addition, the random cellular movement of the monolayers (micromotion) was significantly decreased after odorant exposure. Collectively, these data demonstrate a new physiological role of olfactory receptor signaling in endothelial and epithelial cell barriers and represent a new label-free method to detect odorant binding.

## 1. Introduction

Olfaction on a chip using cells and tissues is showing great promise in detecting and differentiating between thousands of odorants [1,2,3,4]. The microelectrode array (MEA) sensors to date are innovative, but they rely on detecting a change in membrane potential after odorant binding, which may be prone to high false positive rates when transitioning these technologies into the field. Monitoring cell barrier resistance changes after environmental sample addition has been shown in other systems to be a stable and robust field-portable end-point [5,6,7] and thus could contribute valuably to the odorant sensing field.

Olfactory receptors (ORs) are G-protein coupled receptors (GPCRs) that sensitively and specifically bind small, often volatile molecules. In the olfactory epithelium, OR binding to a cognate agonist leads to intracellular signaling through cAMP, which opens cyclic nucleotide-gated Na^+^/Ca^2+^ channels for membrane depolarization that ultimately propagates to odorant sensation in the brain. Interestingly, ORs are expressed in a variety of non-olfactory cells and influence many physiological processes (reviewed in [8]). In non-olfactory cells, intracellular signaling initiated by OR binding can result in increased survival, proliferation, and migration, among other cellular effects, via cAMP or other second messengers, which stimulate diverse signaling pathways [9,10,11]. Even though distinct, physiologically relevant pathways are activated in non-olfactory cells after odorant binding, the olfactory receptor is a GPCR, which initiates signaling via G-proteins. There is a well-established link between different classes of G-proteins and cytoskeletal structure changes that affect cellular morphology [12,13,14] that has not been exploited for odorant sensing.

Cellular impedance via electric cell-substrate impedance sensing (ECIS) technology (Applied BioPhysics Inc., Troy, NY, USA) is a sensitive indicator of cell adhesion and morphology and thus can detect cellular events, including GPCR signaling in real-time [15,16,17]. Thus, we set out to determine whether we could use non-olfactory cells to develop an ECIS-based biosensor to detect odorant binding.

To show proof of concept for detection of odorant binding using an ECIS-based biosensor, two human cell lines (HUVEC and HaCaT) that naturally express ORs were used. The human umbilical vein endothelial cell (HUVEC) line expresses OR10J5, an olfactory receptor that is also naturally expressed in the aorta and coronary artery [18]. Lyral, a ligand of OR10J5, causes HUVEC migration and enhances in vivo angiogenesis, likely via Ca^2+^-dependent protein kinase B (PKB/Akt) signal transduction [18]. The immortalized human keratinocyte (HaCaT) cell line has been shown to express the OR2AT4 olfactory receptor and associated signaling proteins, including the olfactory-specific proteins G-protein alpha subunit (Gα_olf_), adenylate cyclase 3 (ACIII), and Ric8b [10]. In addition, the odorant Sandalore activates HaCaT cells specifically via OR2AT4-cAMP signal transduction to stimulate cell proliferation and migration, processes of wound healing [19].

We hypothesized that odorant binding in non-olfactory cells (HUVECs and HaCaT cells) would cause odorant-specific cytoskeletal rearrangement, leading to morphological changes that could be detected using ECIS. Here we report our results from establishing such an odorant detection system and investigating the types of electrical signals that result from changes in cytoskeletal structure caused by odorant binding in non-olfactory cells.

## 2. Materials and Methods

### 2.1. Cells

Human umbilical vein endothelial cells (HUVECs; American Type Culture Collection (ATCC), Manassas, VA, USA; CRL-1730) were cultured in F-12K (ATCC 30-2204) supplemented with 0.1 mg/mL heparin (#H3393; Sigma-Aldrich Inc., St. Louis, MO, USA), 0.03 mg/mL endothelial cell growth supplement (356006, Corning Inc., New York, NY, USA), and 10% FBS (Corning Inc., New York, NY, USA). Human keratinocytes (HaCaT; AddexBio Technologies, San Diego, CA, USA) were cultured in AddexBio-optimized DMEM (C0003-02) with 10% FBS. Cells were maintained at 37 °C with 5% CO_2_.

### 2.2. Odorants

Lyral (4-(4-Hydroxy-4-methylpentyl)-3-cyclohexene-1-carboxaldehyde) was purchased from Sigma-Aldrich (St. Louis, MO, USA; #95594). Stocks were made in methanol and refrigerated until ready for use. Sandalore^®^ ((3-methyl-5-(2,2,3-trimethyl-1-cyclopent-3-enyl) pentan-2-ol) was purchased from Perfumer Supply House LLC (Danbury, CT, USA; #65113-99-7). Stocks were made in DMSO and refrigerated until ready for use. Odorant stocks were diluted into cell culture media immediately before use, and the final concentration of the diluent (methanol or DMSO) on the cells was 0.1% for all odorant doses tested and the diluent-only controls (0 μM odorant).

In all experiments, cells were exposed to various concentrations of the odorants (0.1–100 μM) as 10× stocks to minimize pipetting disturbances from a complete media change. At least 4 h before the experiment, the media was removed from the cells, and 180 μL of fresh media was added. Next, 20 μL of the 10× odorant stocks was added, and the time course was started.

### 2.3. Electric-Cell Substrate Impedance Sensing (ECIS)

HUVECs (2 × 10^4^ cells/well) or HaCaT cells (4 × 10^4^ cells/well) were seeded in a 96-well ECIS electrode plate (96W10idfPET; Applied Biophysics Inc., Troy, NY, USA) coated with bovine fibronectin (20 μg/mL; Thermo Fisher Scientific Inc., Waltham, MA, USA). Cells were grown for 3 days to form a confluent monolayer. Complex impedance data were obtained using a Z-Theta instrument with a 96-W array station (Applied Biophysics Inc., Troy, NY, USA). Baseline cellular resistance at 4000 Hz was measured in each well for 10–30 min before odorant addition; the reading was then paused while the odorants or controls were added, and resistance readings were resumed. The resistance data were normalized to pre-odorant values to visualize changes and represented as mean ± SEM of one independent experiment representative of three experimental repeats.

To model the resistance data and determine the contribution of R_b_ (paracellular junctions) and α (cell-substrate junctions) to the resistance changes caused by odorant addition, a multiple frequency time (MFT) course ranging from 62.5 to 64,000 Hz was used, and the resulting data were modeled using ECIS^TM^ software (Applied Biophysics Inc., Troy, NY, USA) [20,21,22].

### 2.4. Micromotion

Micromotion was measured by seeding and growing HUVECs or HaCaT cells in a 96-well ECIS electrode plate (96W1E + PET), as described above, to form a confluent monolayer. To quantify micromotion, resistance measurements (4000 Hz) were taken in two wells at intervals of 1 s to provide the data needed to assess quick cellular movements. Thirty minutes of baseline data (without odorant) were obtained, then the resistance measurement was paused, and odorants were added to the cells as 10× treatments to minimize disturbance of the cells. To quantitate the movement in each well, a 1024-point data set was analyzed 30 min after the addition of the odorant. Each data point in the set was first divided by the average of the 1024-point data set. The normalized data set was then separated into groups containing 32 data points, and the variance for each group (Var32) was calculated and averaged to represent the resistance fluctuations with a single number, as described [23,24]. The average Var32 for a treatment well was normalized to the diluent-only control well tested at the same time to yield the Var32 ratio (%). Each Var32 ratio (%) presented in the bar graph is calculated from the average of at least three independent experiments.

### 2.5. cAMP Activity

The measurement of intracellular cAMP levels was performed using the cAMP-Glo^TM^ Assay (Promega^TM^ Corporation, Madison, WI, USA). HUVECs and HaCaT cells (2500 cells/well) were seeded on tissue culture-treated Nunc white MicroWell 96-Well Optical-Bottom Plates (Thermo Fisher Scientific Inc., Waltham, MA, USA) and grown overnight. Cells were exposed to odorants, as detailed above, in serum-free media for 30 min, with a 10 μM forskolin (Thermo Fisher Scientific Inc.) exposure as the positive control. The cells were lysed, and luminescence was analyzed using a microplate-reader (Synergy™ HT, BioTek, Winooski, VT, USA) according to the manufacturer’s protocol. The cAMP activity was represented as cAMP activity relative to controls within each experiment.

### 2.6. Actin and Tubulin Staining

HUVECs (2 × 10^4^ cells/well) or HaCaT cells (4 × 10^4^ cells/well) were seeded into black Nunc 96-MicroWell CC^2^ optical bottom plates with #1.5 borosilicate coverglass base (Thermo Fisher Scientific Inc., Rochester, NY, USA) and grown to confluence.

After odorant exposure, as detailed above, cells used for actin staining were fixed in 3% paraformaldehyde in PBS, permeabilized with 0.5% Triton X-100 and washed with PBS. Block (PBS + 1% BSA) was then added to the cells and allowed to incubate for 1 h. After the blocking step, Alexa Fluor 594 phalloidin (Thermo Fisher Scientific Inc., Waltham, MA, USA; 1:40) was added and incubated for 1 h. Cells were washed with PBS.

After odorant exposure, as detailed above, cells used for tubulin staining were fixed in ice-cold methanol for 3 min and rehydrated with PBS. Alpha-tubulin antibody (Developmental Studies Hybridoma Bank (DSHB) product 12G10, deposited by Frankel, J, and Nelsen, EM) diluted 1:100 in PBS + 1% BSA was added and incubated for 1 h. Cells were washed with PBS and then incubated for one hour after adding a secondary antibody Goat anti-Mouse IgG (H&L)-Alexa Fluor 488 (Thermo Fisher Scientific Inc., Waltham, MA, USA) at 1:100 dilution. Cells were then washed in PBS.

To mount the samples, PBS was removed and one drop of SlowFade™ Gold Antifade Mountant (Thermo Fisher Scientific Inc., Waltham, MA, USA) was added to each well. Cells were viewed on an Olympus IX83 inverted microscope, *Z*-axis equipped, with a DP-80 dual CCD color and monochrome camera. Within each experiment, three images were taken from independent fields of view per treatment condition.

### 2.7. Fluorescence Quantification

Raw TIFF images (16-bit grayscale) were loaded into Fiji for fluorescence quantification. All images were captured using identical microscope exposure/gain settings, and no contrast or brightness changes were made. The mean gray value (the sum of the gray values of all pixels divided by the number of pixels) was analyzed on three replicate images from each odorant concentration within each experiment, and the values were normalized to the controls at each time point. Combined data from three independent experiments are presented as the mean ± SEM.

### 2.8. LDH Release

The measurement of LDH cellular release was performed using the In Vitro Toxicology Assay Kit, Lactic Dehydrogenase based (#TOX7; Sigma-Aldrich, Inc., St. Louis, MO, USA). HUVECs and HaCaT cells were seeded at 2 × 10^4^ and 4 × 10^4^ cells per well, respectively, on tissue culture-treated 96-well plates (Falcon, Corning, NY, USA) and grown for 3 days. Cells were exposed to odorants in reduced serum (1% FBS) media for 1 or 2 h. Hydrogen peroxide (4 mM) exposure was used as a positive control. Absorbance was measured at 490 nm minus background at 690 nm using a microplate-reader (Synergy™ HT, BioTek, Winooski, VT, USA).

### 2.9. Statistics

All data presented were confirmed in at least three independent experiments. Unless noted otherwise, single odorant treatment groups were compared to the control (diluent only) using a two-tailed *t*-test; *p* values ≤ 0.05 (*) or ≤0.01 (**) are indicated.

To examine whether resistance measurements can be used to predict odorant presence, we coded diluent-only controls as 0 and samples with odorant present as 1 and fit a logistic regression (the glm function in R, version 4.1.2). Because resistance measurements are highly correlated across time points, we took the first stable measurement (at 0.5 h) and differences between subsequent time points (resistance at 1 h minus resistance at 0.5 h and similarly between 1.5 and 1 h, and so on until the 2-h time point) as predictors.

A standard five-fold cross-validation (partitioning the data into five groups, or folds) was used to test prediction accuracy. To construct balanced folds, the odorant samples were broken into low, medium, and high concentration bins. Lyral concentration bins were 0.1 to 0.5 μM for low, 1.0 to 10.0 μM for medium, and greater than 10.0 μM for high. Sandalore bins were 0.01 to 0.5 μM for low, 1.0 to 5.0 μM for medium, and greater than 5.0 μM for high. The data were then randomly divided into five groups, each with approximately equal representation of odorant concentration categories (lyral and Sandalore data were analyzed separately). We held back each fold in turn and used the remaining 80% of the samples to fit the logistic regression model. Given estimated regression coefficients and known predictor values for the held back data points, the probability of odorant presence in these samples was estimated. If the *p* values exceeded a cut-off (0.8, 0.85, and 0.9), a given sample was predicted to have the odorant present. Each pass across the folds thus yielded predicted odorant presence or absence for each sample, which were then compared to real values. To explore the reproducibility of these estimates, the random partition of data was repeated 15 times. As measures of prediction accuracy, we used the fraction of negative control samples that were called positive by our model (false positive rate) and the fraction of positive samples (separately in each odorant concentration category) that were called negative (false negative rate).

To determine if the prediction accuracy values could be due to chance, we randomly reshuffled the odorant concentration values. Resistance readings thus now corresponded to random odorant concentration levels, which should eliminate any biologically relevant signal in the data. We then re-ran our model training and prediction. Prediction accuracies obtained from this permuted data set should be entirely due to chance. We expected the prediction accuracies obtained from the actual data to exceed those from the re-arranged data sets if the actual data have predictive power.

## 3. Results and Discussion

### 3.1. Detection of Odorants Using Cellular Resistance

Because odorant binding to olfactory receptors expressed on non-olfactory cells can cause a variety of cellular activities, including cellular contraction, proliferation, and migration [18,19,25,26,27,28], we wanted to determine if electric cell-substrate impedance sensing (ECIS) could be used to detect cell-shape changes that result after odorant binding. As a proof-of-concept, two different cell lines expressing two different olfactory receptors were used: HUVECs express the OR10J5 receptor, which binds to the odorant lyral [18], and HaCaT cells express the OR2AT4 receptor, which binds to the odorant Sandalore [19].

**Figure 1:**
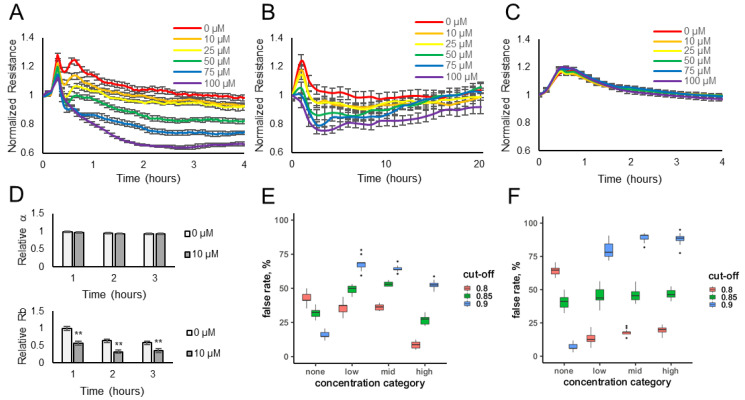
Lyral exposure causes a transient dose-dependent decrease in cellular resistance. HUVEC monolayers (expressing the OR10J5 receptor) were exposed to different concentrations of lyral (10–100 μM), and resistance was monitored over a 4 h (**A**) or 20 h (**B**) period. (**C**) HaCaT monolayers (not expressing the OR10J5 receptor) were exposed to different concentrations of lyral (10–100 μM), and resistance was monitored over a 4 h period. Resistance values (ohms) were normalized to initial readings before odorant addition. Data represented as mean ± SEM of one independent experiment representative of three experimental repeats. (**D**) HUVEC multi-frequency resistance data were modeled to derive parameters, α (basal adhesion) and R_b_ (paracellular barrier), after a 1-, 2-, or 3-h exposure to lyral (0 or 10 μM). Both α and R_b_ values are represented as relative to initial α and R_b_ values before odorant addition. Data shown as mean ± SEM of 3 independent experiments; ** *p* < 0.01 as indicated. Statistical model prediction accuracy using the actual resistance data (**E**) or using permuted (randomly rearranged) resistance data (**F**). The x-axes represent the lyral concentration category: none (0 μM), low (0.1 to 0.5 μM), medium (1.0 to 10.0 μM), and high (greater than 10.0 μM), and the y-axes shows the false rate percentage, reflecting false positive or negative rates depending on the x-axes categories. Samples in the none category but mis-predicted as containing odorant are false positives. Samples in the low, medium, or high category but do not contain odorant are the false negative rate, also plotted on the y-axes.

**Figure 2:**
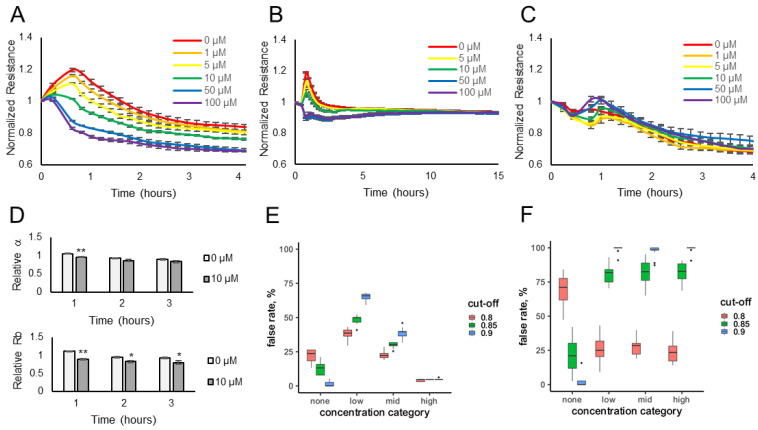
Sandalore exposure causes a transient dose-dependent decrease in cellular resistance. HaCaT monolayers (expressing the OR2AT4 receptor) were exposed to different concentrations of Sandalore (1–100 μM), and resistance was monitored over a 4 h (**A**) or 15 h (**B**) period. (**C**) HUVEC monolayers (not expressing the OR2AT4 receptor) were exposed to different concentrations of Sandalore (1–100 μM), and resistance was monitored over a 4 h period. Resistance values (ohms) were normalized to initial readings before odorant addition. Data represented as mean ± SEM of one independent experiment representative of three experimental repeats. (**D**) HaCaT multi-frequency resistance data were modeled to derive the parameters, α (basal adhesion) and R_b_ (paracellular barrier), after a 1-, 2-, or 3-h exposure to Sandalore (0 or 10 μM). Both α and R_b_ values are represented as relative to initial α and R_b_ values before odorant addition. Data shown as mean ± SEM of 3 independent experiments; ** *p* < 0.01 and * *p* < 0.05 as indicated. Statistical model prediction accuracy using the actual resistance data (**E**) or permuted (randomly rearranged) resistance data (**F**). The x-axes represent the Sandalore concentration category: none (0 μM), low (0.01 to 0.5 μM), medium (1.0 to 5.0 μM), and high (greater than 5.0 μM), and the y-axes show the false rate percentage, reflecting false positive or negative rates depending on the x-axes categories. Samples in the none category but mis-predicted as containing odorant are false positives. Samples in the low, medium, or high category but that do not contain odorant are the false negative rate, also plotted on the y-axes.

Electrical resistance values were measured over time for HUVECs or HaCaT cells exposed to various concentrations of the odorants (10–100 μM) or diluent control (no odorant). The data show that the resistance values (normalized to the pre-odorant values) decreased in a dose-dependent manner when lyral was added to HUVEC monolayers (Figure 1A) or when Sandalore was added to HaCaT monolayers (Figure 2A). The observed response was transient, as resistance values return to normal after 15–20 h (Figure 1B and Figure 2B). The transience of the response may relate to the volatility of the odorants or to odor adaptation, which may involve desensitization of the signaling pathway and/or internalization of the odorant receptor as documented in olfactory systems [29,30].

To determine if the resistance decrease observed was due to odorant binding and not a non-specific cellular event, lyral (10–100 μM) was added to HaCaT cells, which do not express the OR10J5 receptor [10], and Sandalore (1–100 μM) was added to HUVECs, which have not been reported to express the OR2AT4 receptor, and in either case normalized resistance values did not change over 4 h (Figure 1C and Figure 2C, respectively). Cellular resistance values can also decrease from cytotoxicity; however, this is unlikely because the effect is transient (Figure 1B and Figure 2B), and an LDH release assay was performed and showed no changes in LDH release even after the cells were exposed to the highest dose of the odorants (100 μM) for 2 h (Figure S1A,B).

The cellular resistance changes after odorant addition were modeled using ECIS software. R_b_ (in cm^2^ × ohm) describes the resistance of cell–cell contacts to current flow, and α (in cm × ohm^0.5^) describes resistance of cell-electrode contacts to current flow [20,21,22]. Complex impedance data obtained over a range of frequencies from HUVEC monolayers treated with diluent only (0 μM) or 10 μM lyral were modeled and revealed that R_b_ values, not α values, significantly changed after the addition of lyral (Figure 1D), revealing that odorant addition causes a decrease in cell–cell contacts. When the same modeling was performed with HaCaT monolayers treated with diluent only (0 μM) or 10 μM Sandalore, both R_b_ and α values significantly decreased after a 1-h exposure to Sandalore (Figure 2D). However, longer exposures (2–3 h) to Sandalore caused only a decrease in R_b_ values, revealing that odorant addition caused an initial decrease in both cell-matrix and cell–cell contacts, with a longer-term decrease only in cell–cell contacts. Lower concentrations of the odorants (0.01–10 μM) were also evaluated (Figure S2A,B) to aid in the fitting of a statistical model. All resistance data obtained after odorant exposure were used to fit a statistical model that discriminates between odorant presence and absence, based on data where sample identity is known a priori. Resistance measurements from unknown samples can then be fed into the model, reading out probability of odorant presence and thus automating detection for future sensing applications. A cross-validation approach was used to evaluate the prediction accuracy of ECIS resistance changes for odorant detection.

A stringent cut-off (*p* > 0.9) for lyral detection yielded a 54% false positive rate (Figure 1E), with a 15% false negative rate at the high concentration of lyral. Relaxing the stringency resulted in a substantial elevation of the false positive rate. Nevertheless, false negative rates, even at low concentrations of this odorant, were substantially below those expected by chance (85%, Figure 1F).

A stringent cut-off (*p* > 0.9) for Sandalore detection yielded no false positives, with a low (6%) false negative rate among samples with high (5 μM or greater) concentrations of the odorant (Figure 2E). We failed to detect intermediate Sandalore concentrations (between 0.5 and 5 μM) 40% of the time, and low (less than 0.5 μM) concentrations in 64% of the samples. However, these false positive rates for intermediate and low concentrations were still substantially lower than those expected by chance (Figure 2F) and could be reduced almost by half by adopting a less stringent cut-off (*p* > 0.85), albeit at the price of elevating the false negative rate to 16%.

Overall, it appears that our approach has promise for automating the detection of odorants in unknown environmental samples, as well as for deorphanizing ectopically expressed olfactory receptors, and testing environmental samples or odorant candidates in a high-throughput format. Notably, our prediction accuracy was degraded by pooling controls across different experiments performed on different days; this was especially true for the HUVEC monolayers, which showed variability between controls on different days. Future modeling efforts will pair controls with experimental samples for high prediction accuracy. The initial resistance disturbances observed in all experiments is because the cells must be removed from the incubator and the odorants are hand pipetted onto each well. Further, transitioning this cell-based biosensor from an open-well pipetting platform to an automated enclosed fluidic chip will decrease the variability in the controls, as we have previously reported for another ECIS-based cell sensor [5].

### 3.2. Determination of cAMP Activity after Odorant Exposure

**Figure 3:**
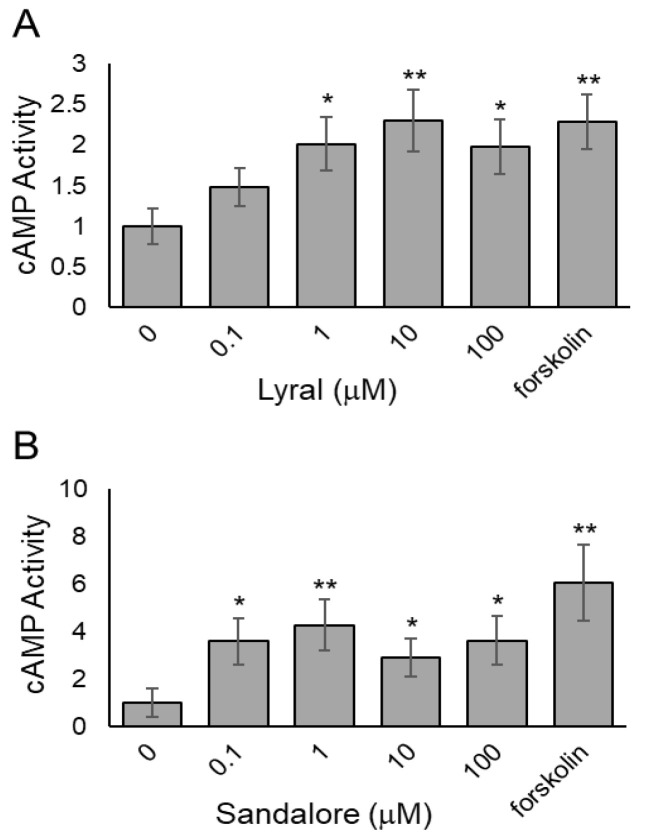
Odorants induce cAMP accumulation in cell monolayers. (**A**) HUVEC monolayers were exposed to different concentrations of lyral (0.1–100 μM) or forskolin (10 μM) as a positive control, and cAMP was measured after 30 min. (**B**) HaCaT monolayers were exposed to different concentrations of Sandalore (0.1–100 μM) or forskolin (10 μM) as a positive control, and cAMP was measured after 30 min. Data were normalized to controls exposed to diluent only (0 μM). The data are shown as mean ± SEM of 3 independent experiments; ** *p* < 0.01 and * *p* < 0.05 as indicated.

Other researchers have shown an odorant-induced activation of the cAMP-Ca^2+^ signaling pathway in non-olfactory cells [19,31,32,33], reminiscent of the pathway in olfactory sensory neurons leading to cyclic nucleotide-gated ion channel opening. To determine if odorant binding in our cellular system activates the olfactory receptors and causes olfactory signaling, the intracellular second messenger, cAMP, was measured after odorant exposure. Briefly, HUVECs (Figure 3A) or HaCaT cells (Figure 3B) were seeded, and 24 h later, the cells were exposed to various concentrations of lyral (0.1–100 μM) or Sandalore (0.1–100 μM), respectively. After a 30-min exposure, the cells were lysed, and intracellular cAMP levels were measured. Both HUVECs (Figure 3A) and HaCaT cells (Figure 3B) exhibited a significant increase in intracellular cAMP levels, suggesting the odorants are binding to cell surface olfactory receptors and causing activation of intracellular signaling pathways. The cAMP dose response in HaCaT is similar in magnitude to previously reported work [19]. A cAMP response has not been documented in HUVECs after lyral exposure, although a dose-dependent increase in Ca^2+^ has been observed [18].

### 3.3. Characterization of Cytoskeletal Organization after Odorant Exposure

**Figure 4:**
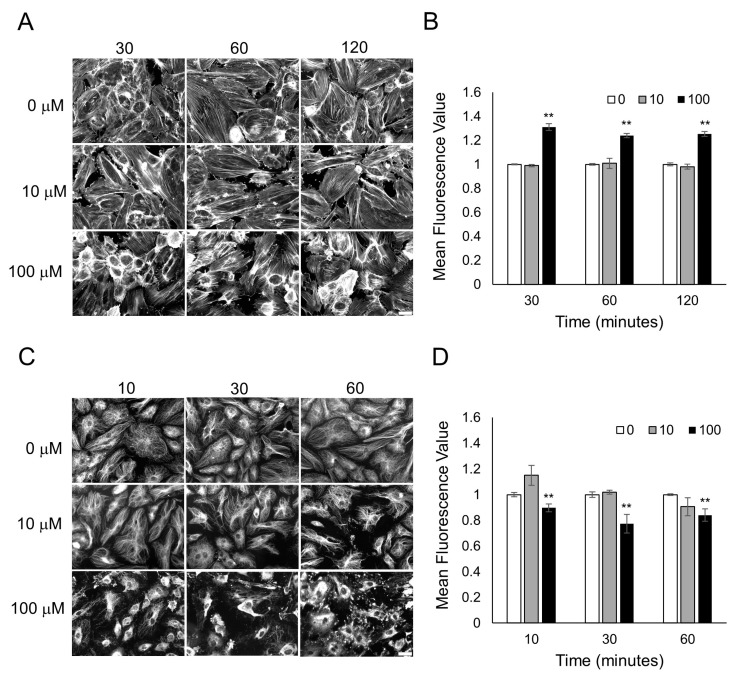
Lyral exposure causes F-actin assembly and microtubule disassembly in HUVECs. Fluorescent images of F-actin phalloidin staining (**A**) and tubulin staining (**C**) after HUVECs were treated with 0, 10, 100 μM lyral for 10–120 min. Scale bar = 50 μm. Quantitative mean fluorescence intensity of F-actin staining (**B**) and tubulin staining (**D**) was measured using Fiji. A two-tailed *t*-test was performed to compare each lyral-treated group to the control group completed at the same time point; ** *p* < 0.01 as indicated.

To examine why odorant binding in endothelial and epithelial monolayers is causing a decrease in cellular resistance caused primarily by a decrease in cell–cell junctions, we examined the cytoskeleton of the cells after odorant exposure. Actin microfilaments and microtubules (tubulin polymers) in endothelial and epithelial monolayers are connected to tight and adherens junctions (AJs) and play critical roles in cell barrier functions [34,35,36,37,38]. To determine if odorant binding could change the actin microfilaments or the tubulin-containing microtubules, HUVECs were exposed to different concentrations of lyral (10 or 100 μM), and F-actin and microtubule organization was examined over time. When HUVECs were exposed to diluent only (0 μM lyral), the actin in the cell monolayers was organized as a mixture of cortical rim and stress fibers, but after exposure to 100 μM lyral, a notable increase in gaps between the cells (large dark areas) and an increase in cortical rim actin was observed (Figure 4A). Exposure to an intermediate dose of lyral (10 μM) causes a noticeable increase in gaps between cells, but did not cause an increase in cortical rim actin. Quantification of mean fluorescence intensity from triplicate images from three independent experiments indicates that the highest dose of lyral (100 μM) caused a significant increase in the assembly of F-actin (polymerized actin) in HUVECs (Figure 4B). In addition, when HUVECs were exposed to diluent only (0 μM lyral), the tubulin in the cell monolayers was a finely dispersed network of microtubules, but exposure to lyral caused disassembly of the tubulin-containing microtubules, as shown in Figure 4C and quantitated in Figure 4D.

**Figure 5:**
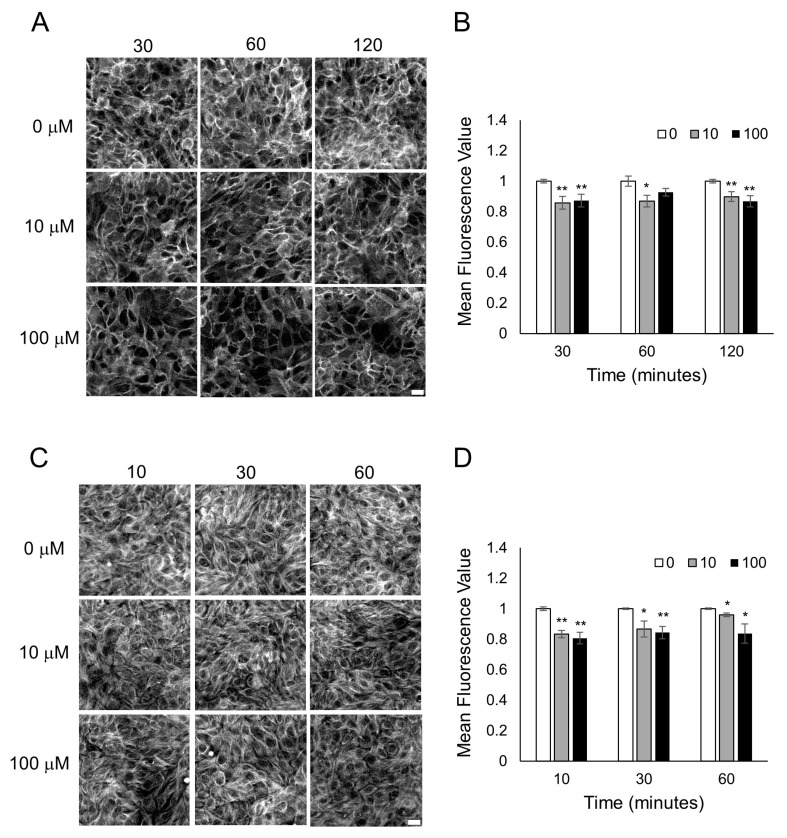
Sandalore exposure causes F-actin and microtubule disassembly in HaCaT cells. Fluorescent images of F-actin phalloidin staining (**A**) and tubulin staining (**C**) after HaCaT cells were treated with 0, 10, 100 μM Sandalore for 10–120 min. Scale bar = 20 μm. Quantitative mean fluorescence intensity of F-actin staining (**B**) and tubulin staining (**D**) was measured using Fiji. A two-tailed *t*-test was performed to compare each lyral-treated group to the control group completed at the same time point; ** *p* < 0.01 and * *p* < 0.05 as indicated.

HaCaT cells were also examined to determine if Sandalore exposure could change actin microfilaments or tubulin-containing microtubules in epithelial cell monolayers. When HaCaT cells were exposed to diluent only (0 μM Sandalore), the actin in the cell monolayers was mainly present in the cortical rim, and after exposure to 10 or 100 μM lyral, a notable decrease in cortical rim actin was observed (Figure 5A). Small patches of F-actin disassembly were observed, which was not uniform throughout the cell layer. Quantification of mean fluorescence intensity from triplicate images from three independent experiments indicates that Sandalore exposure caused a significant F-actin decrease in HaCaT cells (Figure 5B). When HaCaT cells were exposed to diluent only (0 μM Sandalore), tubulin in the cell monolayers was in a finely dispersed network of microtubules, but exposure to Sandalore caused disassembly of small patches of tubulin, as shown in Figure 5C and quantitated in Figure 5D.

Interestingly, the odorants are causing disassembly of the microtubules in both cell systems but have a discordant effect on F-actin organization. Microtubules and actin microfilaments have been shown to play an essential role in the barrier function of both endothelial and epithelial monolayers [37,39,40,41], but microtubule disassembly does not always lead to disassembly of cortical band actin during cell barrier dysfunction. For example, thrombin causes the disassembly of microtubules and early actin stress fiber formation, followed by cortical actin accumulation and cell barrier dysfunction [40,42]. Therefore, the two odorants may have a differing effect on the cytoskeleton because olfactory signal transduction pathways in non-olfactory epithelial and endothelial cells are very diverse [11].

Odorant binding in both cell lines is causing an increase in cAMP, and the organization of the cytoskeleton is strongly dependent on both cAMP levels and localization within cells. Normally an increase in cAMP promotes actin depolymerization [12]. This association is borne out in HaCaT monolayers treated with Sandalore, where we observe both a significant increase in cAMP and a decrease in F-actin. Interestingly, this association was not observed in HUVEC monolayers treated with lyral. In HUVECs, lyral binding caused a smaller increase in cAMP and an increase in F-actin in the cortical rim. However, other reports indicate that not only is the level of cAMP important in downstream events, but cAMP subcellular localization can drastically change, resulting cellular activities [43]. For example, increases in soluble cAMP (not membrane-bound cAMP) can disrupt the cell barrier without a decrease in cortical band actin [37]. Our cAMP data do not differentiate between cytosolic and membrane-bound cAMP because they are based on whole cell lysates. Additionally, G protein-coupled receptors (GPCR) do not only signal through transmembrane adenylyl cyclases, but can activate soluble adenylyl cyclases, leading to an increase in soluble cAMP pools [44]. Thus, a more in-depth signaling analysis after lyral and Sandalore binding may reveal distinct signaling mechanisms in these two cell types.

### 3.4. Detection of Odorants Using Cellular Micromotion

Based on previous reports showing that cellular micromotion depends on the dynamics of the cytoskeleton [20,24,45,46,47], combined with our data showing that the odorants change the structure of the cytoskeleton, we hypothesized that the measurement of the cellular micromotion (fluctuations in electrical resistance measurements over time) would be an additional method to detect odorant binding.

To measure micromotion in HUVEC and HaCaT cell monolayers, cells were grown to confluence on 96-well ECIS electrode plates over three days, fed 18–24 h before the experiment, and then resistance (at 4000 Hz) was monitored after various concentrations of odorants (1–100 μM) or diluent control (0 μM) were added to the cells. Resistance readings were obtained every second in each well to facilitate quick motion measurements. To quantify the movement in each well, a 1024-point resistance data set was collected 30 min after odorant addition to minimize micromotion variations due to pipetting and to allow time for post-odorant signal transduction and cytoskeletal changes to take place. The 1024-point resistance data set was separated into 32 groups of 32 data points, and the variance within each group (Var32) was calculated, as fully described in the methods. The average Var32 for each treatment well was then normalized to the control well completed at the same time, and at least three independent experiments were averaged together to yield the Var32 ratio (%).

**Figure 6:**
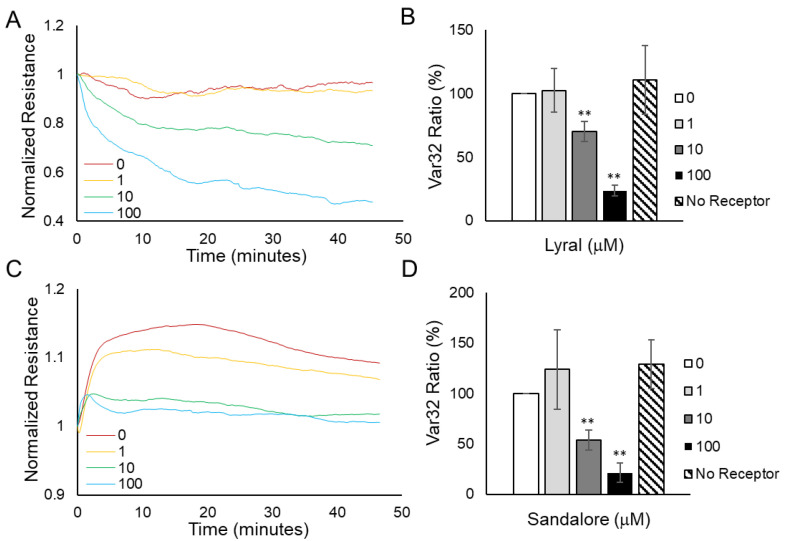
Odorant exposure causes a dose-dependent decrease in cellular micromotion. HUVEC monolayer (**A**) and HaCaT monolayer (**C**) resistance data obtained every second after various concentrations of odorants were added. Var32 analysis of (**B**) HUVEC normalized resistance data 30 min after lyral addition or (**D**) HaCaT normalized resistance data 30 min after Sandalore addition. The average Var32 for each treatment well was normalized to the control well tested at the same time, and at least three independent experiments were performed and averaged together to represent the Var32 ratio (%) presented as mean ± SEM. The analysis shows a significant decrease in the micromotion of both HUVEC and HaCaT monolayers after 10 or 100 μM of lyral or Sandalore, respectively, was added. As an additional negative control, a 100 μM dose of the odorant was added to a cell monolayer that did not express the corresponding receptor (no receptor) and no significant decrease in micromotion was observed. A two-tailed *t*-test was performed to compare each odorant-treated group to the control; ** *p* < 0.01 as indicated.

Figure 6A shows a graph of HUVEC monolayer resistance data obtained every second after various concentrations of lyral were added. Notably, small fluctuations in the resistance readings when 0 μM or 1 μM lyral were added decreased with higher doses of lyral (10 μM or 100 μM lyral). Quantification of HUVEC micromotion (Figure 6B) reveals that higher doses of lyral (10 μM or 100 μM) cause a significant decrease in micromotion. As an additional control, 100 μM lyral was added to HaCaT cells (no OR10J5 receptor), and no significant decrease in micromotion was observed.

Figure 6C shows a graph of HaCaT cell monolayer resistance data obtained every second after various concentrations of Sandalore were added. Unlike the HUVEC resistance values shown in Figure 6A, it is difficult to visually observe the resistance fluctuations in HaCaT monolayers. The lack of visual resistance fluctuations was not unexpected because HaCaT cells form a tight epithelial barrier compared to HUVECs, which form a more dynamic endothelial barrier. Interestingly, the average Var32 value before normalization in control HaCaT monolayers was 1.6 × 10^−7^, while in HUVEC monolayers, the average value was 10-fold higher at 7.6 × 10^−6^, indicating that HUVEC monolayers move more than HaCaT monolayers. Even though the HaCaT monolayer resistance fluctuations were small, quantification of the micromotion data (Figure 6D) revealed that higher doses of Sandalore (10 μM or 100 μM) caused a significant decrease in micromotion. As an additional control, 100 μM Sandalore was added to HUVECs (no OR2A4 receptor), and no significant decrease in micromotion was observed.

Our observed ECIS micromotion changes corroborate with prior work in epithelial cells showing that both actin and tubulin dynamics contribute to changes in micromotion measured using ECIS [47]. In our experiments, lyral binding to the OR10J5 caused both actin polymerization and microtubule depolymerization, and Sandalore binding to OR2AT4 caused actin and microtubule depolymerization, which lead to measurable changes in micromotion. A linkage between odorant binding, cAMP, and changes in micromotion has been documented in the literature. We observed an increase in cAMP after odorant binding and a decrease in micromotion, and interestingly, fibroblasts treated with a cAMP activator, 8-br-cAMP, exhibited less micromotion than untreated cells [48]. Furthermore, odorant binding in human airway smooth muscle cells inhibited the spontaneous cytoskeletal motions of the cells as measured by examining trajectory maps of unforced ferrimagnetic beads bound to the cells [26], and may be the basis for the phenotypic change in the cells predisposing the airways to hyperplasia and asthma. The physiological significance of a decreased cell micromotion observed in this study in both cells 30 min after odorant binding is unknown but may be related to the early changes occurring before the odorant-induced angiogenesis observed in HUVECs [18] or the odorant-induced wound healing described in HaCaT cells [19].

## 4. Conclusions

The present study is the first to demonstrate that olfactory receptor signaling alters the cytoskeleton, leading to changes in the barrier function and micromotion of endothelial and epithelial cell monolayers. Using resistance to measure cell barrier function and micromotion are novel cellular endpoints that have not been previously described in odorant sensing. Furthermore, these odorant-induced cellular responses occurred rapidly (under 1 h) and are sensitive to μM amounts of odorants. This cellular sensor may be used repeatedly because the resistance changes are transient. In addition, olfactory receptors can be transfected into non-olfactory cells such as HEK293 and HeLa cells (reviewed in [49], potentially developing this technology into a multiplexed, low-cost, real-time odorant sensor. High-throughput transcriptional profiling to deorphanize ORs by two groups have reported differences in detection outcomes [50,51]; thus, having diverse end-point assays to contribute and validate the olfactory receptor binding is essential. In addition, electrically assessing barrier function in epithelial and endothelial cells has already been validated as a robust, stable, and field portable endpoint [5,6,7]. Therefore, monitoring the changes in the integrity and motion of cell barriers after odorant binding will be important as monitoring platforms become more commonplace in field applications and high-throughput laboratory-based assays.

## Data Availability

Not applicable.

## Supplementary Materials

The following supporting information can be downloaded at: https://www.mdpi.com/article/10.3390/bios13030329/s1, Figure S1: Odorant exposure does not decrease HUVEC or HaCaT cell viability. (A) HUVECs were exposed to 100 μM lyral or 4 μM hydrogen peroxide (positive control) for 1 or 2 h, and lactate dehydrogenase (LDH) release was assayed. (B) HaCaT cells were exposed to 100 μM Sandalore or 4 μM hydrogen peroxide (positive control) for 1 or 2 h, and lactate dehydro-genase (LDH) release was assayed. Data represented as mean ± SEM of three independent ex-periments. A two-tailed *t*-test was performed to compare each treated group to control; ** *p* < 0.01 as indicated; Figure S2: Low dose odorant exposures. (A) HUVEC monolayers were exposed to different concentrations of lyral (0.1–10 μM) and resistance was monitored over a 4 h period. (B) HaCaT monolayers were exposed to different concentrations of Sandalore (0.01–5 μM) and resistance was monitored over a 4 h period. Data represented as mean ± SEM of one independent experi-ment representative of three experimental repeats.
