# Supplementary material for: Odorant Binding Causes Cytoskeletal Rearrangement, Leading to Detectable Changes in Endothelial and Epithelial Barrier Function and Micromotion
Source: Biosensors (Basel). 2023 Feb 28;13(3):329. doi: 10.3390/bios13030329 (PMC10046532; doi:10.3390/bios13030329)
Supplement: Supplementary file 1 [file biosensors-13-00329-s001.zip › biosensors-2230420-supplementary.pdf]

biosensors

MDPI

Supplementary Materials

Odorant Binding Causes Cytoskeletal Rearrangement, Leading to Detectable Changes in Endothelial and Epithelial Barrier Function and Micromotion

Theresa M. Curtis [1,*], Annabella M. Nilon [1], Anthony J. Greenberg [2], Matthew Besner [1], Jacob J. Scibek [1], Jennifer A. Nichols [3] and Janet L. Huie [3]

[1] Department of Biological Sciences, SUNY Cortland, Cortland, NY 13045, USA
[2] Bayesic Research, LLC, Ithaca, NY 14850, USA
3 Jan Biotech, Inc., Ithaca, NY 14850, USA
* Correspondence: theresa.curtis@cortland.edu

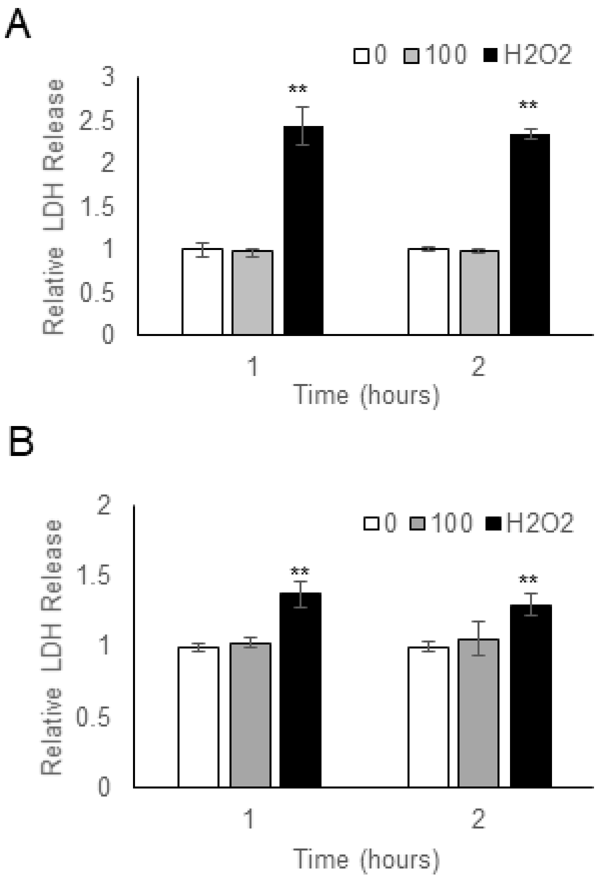

Figure S1. Odorant exposure does not decrease HUVEC or HaCaT cell viability. (**A**) HUVECs were exposed to 100 µM lyral or 4 µM hydrogen peroxide (positive control) for 1 or 2 hours, and lactate dehydrogenase (LDH) release was assayed. (**B**) HaCaT cells were exposed to 100 µM Sandalore or 4 µM hydrogen peroxide (positive control) for 1 or 2 hours, and lactate dehydrogenase (LDH) release was assayed. Data represented as mean ± SEM of three independent experiments. A two-tailed *t*-test was performed to compare each treated group to control; ** $p < 0.01$ as indicated.

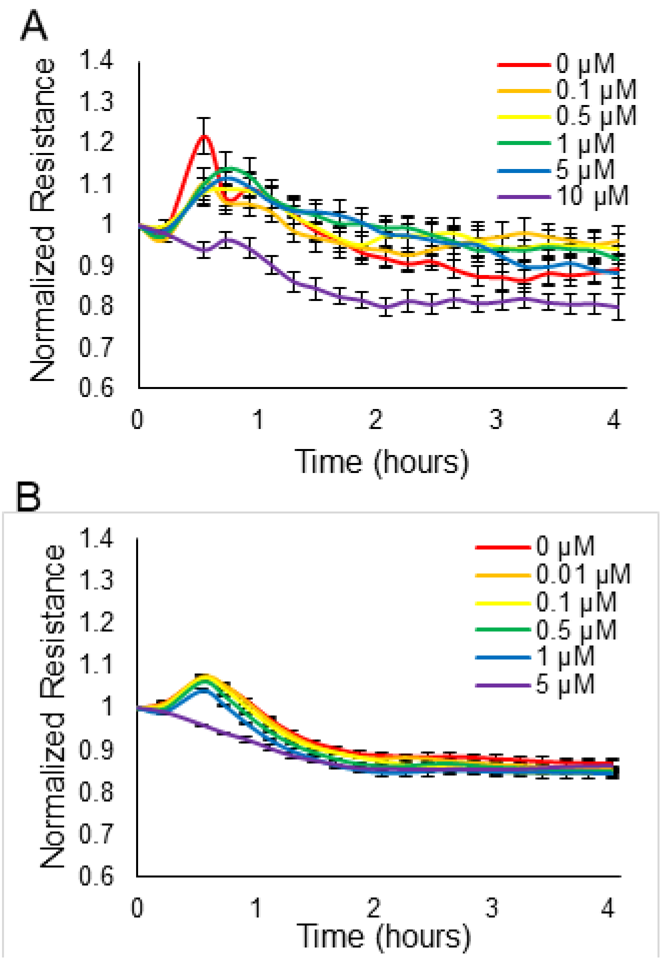

Figure S2. Low dose odorant exposures. (**A**) HUVEC monolayers were exposed to different concentrations of lyral (0.1–10 μM) and resistance was monitored over a 4 h period. (**B**) HaCaT monolayers were exposed to different concentrations of Sandalore (0.01–5 μM) and resistance was monitored over a 4 h period. Data represented as mean ± SEM of one independent experiment representative of three experimental repeats.
